# Transforming electronic health record polysomnographic data into the Observational Medical Outcome Partnership's Common Data Model: a pilot feasibility study

**DOI:** 10.1038/s41598-021-86564-w

**Published:** 2021-03-29

**Authors:** Jeong-Whun Kim, Seok Kim, Borim Ryu, Wongeun Song, Ho-Young Lee, Sooyoung Yoo

**Affiliations:** 1 Department of Otorhinolaryngology, Seoul National University College of Medicine, Seoul, Republic of Korea; 2 Department of Otorhinolaryngology, Seoul National University Bundang Hospital, Seongnam, Republic of Korea; 3 Office of eHealth Research and Business, Seoul National University Bundang Hospital, Seongnam, Republic of Korea

**Keywords:** Medical research, Risk factors

## Abstract

Well-defined large-volume polysomnographic (PSG) data can identify subgroups and predict outcomes of obstructive sleep apnea (OSA). However, current PSG data are scattered across numerous sleep laboratories and have different formats in the electronic health record (EHR). Hence, this study aimed to convert EHR PSG into a standardized data format—the Observational Medical Outcome Partnership (OMOP) common data model (CDM). We extracted the PSG data of a university hospital for the period from 2004 to 2019. We designed and implemented an extract–transform–load (ETL) process to transform PSG data into the OMOP CDM format and verified the data quality through expert evaluation. We converted the data of 11,797 sleep studies into CDM and added 632,841 measurements and 9,535 observations to the existing CDM database. Among 86 PSG parameters, 20 were mapped to CDM standard vocabulary and 66 could not be mapped; thus, new custom standard concepts were created. We validated the conversion and usefulness of PSG data through patient-level prediction analyses for the CDM data. We believe that this study represents the first CDM conversion of PSG. In the future, CDM transformation will enable network research in sleep medicine and will contribute to presenting more relevant clinical evidence.

## Introduction

Obstructive sleep apnea (OSA) is an significant risk factor in several major health conditions, such as cardiovascular^1–4^, neurovascular^5,6^, and metabolic diseases^7,8^. OSA is diagnosed on the basis of certain crucial parameters, including the apnea–hypopnea index (AHI) of polysomnography (PSG). PSG is a standard diagnostic sleep test for OSA, and its results hold significant clinical implications for various major diseases. For example, severe OSA with AHI > 30 is known to be correlated with the development of strokes and incident hypertension^5,9^. Although large-scale prospective cohort studies can be used to empirically prove such important clinical observations, they suffer from the limitations of long follow-up periods and high costs. On the other hand, retrospective studies can only establish statistical associations between the risks of major conditions and PSG results rather than a definitive causal relationship. Furthermore, PSG is a whole-night test, and the capacity for PSG tests per sleep center is thus limited. However, multi-center collaborative studies can be used to conduct more PSGs, and well-defined large-volume PSG databases have the potential to corroborate the validity of conjectured correlations. The analysis of a wide range of electronic health record (EHR) data, including medical conditions, drug exposures, procedures, and measurements, in conjunction with PSG data, and their rapid verification across multiple institutions may enable the procurement of crucial pieces of robust scientific evidence through enhancements in analytic power.

However, as the primary goal of EHR is medical application, rather than research, the reuse of EHR data for academic purposes necessitates the mapping of clinical observations to standard vocabularies^10^. To this end, Observational Health Data Sciences and Informatics (OHDSI), an international collaborative initiative, has created and applied an open-source standard data format and analytic solutions to diverse health and medical databases across the world^11^. The Observational Medical Outcome Partnership's (OMOP) common data model (CDM), which is utilized by OHDSI as a standard data format, serves as a guide for the standardization of heterogeneous representations of healthcare data obtained from disparate sources. Conversion of health and medical databases into the CDM format is expected to enable interdisciplinary collaborative large-scale analyses. Such large-scale analyses using open-source analytic tools based on standardized datasets are, in turn, expected to improve the speed and efficiency of population-level estimation and patient-level prediction, thereby enhancing the reliability of clinical decision-making^11,12^.

To the best of our knowledge, measurements obtained via PSG are yet to be transformed into the CDM format. Linking the diverse data obtained from PSG with the extensive EHR database in a structured CDM format is expected to facilitate multi-center studies and strengthen general analytic power. In this study, we aimed to convert EHR PSG data into the standardized OMOP CDM data format and conduct a pilot feasibility test. Through a pilot feasibility study, we attempted to confirm the possibility of developing a predictive model using existing CDM data and additional PSG data, and to verify the usefulness of the integrated data.

## Methods

### Study population for CDM conversion

This study included patients who visited the Sleep Center at Seoul National University Bundang Hospital (SNUBH), located in the metropolitan area of Seoul in South Korea, and had undergone PSG between February 2004 and June 2019.

### Data source

OMOP CDM data obtained from SNUBH were used in this study. In particular, the data comprised de-identified EHR data based on OMOP CDM version 5.3.1 and accumulated over a period of 16 years—from the opening of SNUBH with the full EHR system in May 2003, till June 2019. The EHR data of more than 2 million patients, including patient demographics, diagnosis, chief complaints, drug exposures, test orders/results, vital signs, surgeries, family histories, and past medical histories, were converted to CDM.

This study was performed in accordance with the relevant guidelines and regulations of the SNUBH Institutional Review Board (IRB) and was approved by the SNUBH IRB. As it is an observational study and the data source was de-identified, this study was approved based on waivers of informed consent or exemptions by the SNUBH IRB (IRB No: X-2002–592-904).

### Polysomnographic parameters

We considered all PSGs performed at the Sleep Center of SNUBH as target data to be converted into OMOP CDM, including full-night PSGs, split-night PSGs, PSGs for continuous positive airway pressure (CPAP) titration, and multiple sleep latency tests (MSLTs). In the case of split-night PSGs, the values of the parameters represented only the diagnostic portions in this study. No home sleep apnea tests were included because they are not popular in South Korea. The PSG parameters to be transformed into OMOP CDM included information related to sleep architecture, respiratory activity, positions during sleep, blood oxygen saturation, and limb movement.

We conducted PSGs using an Embla N 7000 (Embla, Reykjavik, Iceland) recording system equipped with standard electrodes and sensors, in the presence of a sleep technician. The entire PSG retinue consisted of electroencephalography, electrooculography, echocardiography, submental and limb electromyography, chest and abdominal plethysmography, nasal pressure manometry, oronasal thermistor, pulse oximetry, and a snoring sensor. Apnea was defined as a pause in the respiratory airflow lasting at least 10 s, and hypopnea was defined as a reduction in the airflow by 50% or more lasting at least 10 s, or the accompaniment of airflow reduction by arousal or an oxygen desaturation by 4% or more^13^. The PSG data were reviewed and scored by sleep experts using the Embla RemLogic PSG Software (Embla, ON, Canada). The study report from the Embla RemLogic PSG Software has the following parameter (variable) categories: patient information; sleep summary; summary graph; sleep information; arousal statistics; autonomic arousal (plethysmogram) statistics; apnea/hypopnea statistics; apnea-desaturation relation; Cheyne Stokes breathing statistics; breath statistics; snoring statistics; flattening statistics; respiratory mechanic instability statistics; SpO2 statistics; desaturation statistics; heart rate statistics; cardiac events; bruxism; rapid eye movement sleep behavior disorder information; rhythmic movement disorder information; periodic limb movement statistics; and position statistics. Among them, the sleep experts at our sleep center selected the PSG parameters that are commonly employed in the literature to make available in the PSG summary report of our EHR. The selected parameters were automatically exported and imported into our EHR in a structured format.

### Strategy to convert PSG data into OMOP CDM

We designed and implemented the following extract–transform–load (ETL) process to transform the PSG data into the OMOP CDM format.

Despite being reported in a structured form, the EHR PSG results considered in this study had been revised approximately 11 times. Hence, we extracted the data corresponding to each revised form and integrated them within the CDM format via standardization. The procedural information for PSG order itself had already been converted into the CDM format. Thus, in this study, we linked the extracted PSG results and the corresponding existing orders in the CDM to connect the PSG procedures with their corresponding results.

The PSG parameters were manually mapped by sleep domain experts (J.-W. Kim and S.-W- Cho) to standard concepts within the Logical Observation Identifiers Names and Codes (LOINC) or Systematized Nomenclature of Medicine–Clinical Terms (SNOMED CT) vocabularies corresponding to the *measurement* and *observation* domains. Non-mapped parameters were added to the *concept*, *concept_ancestor*, and *concept_relationship* tables to be used as new custom standard concepts (please see Supplementary Table S1 for the concept mapping information in the case of PSG and Supplementary Table S2 for the concept definitions). More than 2 billion digits were assigned to the *concept_id* of the new custom concepts. In the *concept_ancestor* table, the newly added concepts served as their own ancestors and descendants. In the *concept_relationship* table, the mapping information between source and standard concepts was added. Additionally, we described the bidirectional relationship between PSG and its parameters in the table using the concepts of ‘*Panel contains*’ and ‘*Contained in panel (LOINC)*’ relationships.

**Figure 1:**
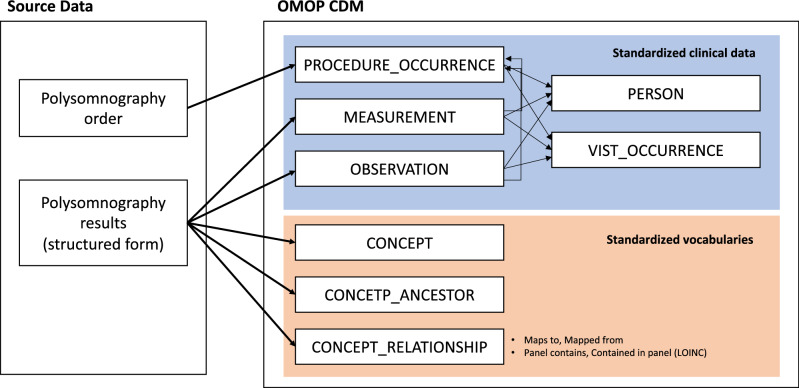
Conversion of polysomnography into the Observational Medical Outcomes Partnership (OMOP) Common Data Model (CDM) tables.

The extracted PSG data were transformed and loaded into *measurement* and *observation* tables with standard concepts. Observation data were linked to the corresponding PSG procedures via the *observation_event_id* and *obs_event_field_concept_id* fields. In order to link measurements with corresponding procedures, we used the new *modifier_of_event_id* and *modifier_of_field_concept_id* fields that have been proposed by the OHDSI Oncology Working Group^14^. The *procedure_occurrence*, *measurement*, and *observation* tables were linked to the person and *visit_occurrence* tables based on their foreign keys. The CDM tables associated with the PSG data are depicted in Fig. 1.

After completing the ETL, we assessed the PSG data quality via exploratory data analysis and developed data quality check rules for data cleaning (please see Supplementary Table S3 for the detailed cleaning rules and the number of records filtered by the rules). Finally, the cleaned PSG data integrated into the existing CDM were utilized for a feasibility test.

### Pilot feasibility test using open-source OHDSI analytic tools

We conducted a pilot feasibility test using only full-night PSG tests of patients 18 years or older. The feasibility test was designed to develop and validate a model to predict cardio-neuro-metabolic disease within a target population between a period of 1 day and 1095 days from the target cohort start date of the PSG test. A cardio-neuro-metabolic disease was defined as any condition involving International Classification of Disease, Tenth Revision (ICD-10) codes corresponding to the comorbidities listed in Supplementary Table S4. We included any occurrence of the defied ICD-10 codes without constraints on the frequency.

In the population setting for the patient-level prediction, varying minimum lookback periods of 30 days, 90 days, and 180 days were utilized for the prior observation periods of patients from the target population. Subjects without time-at-risk of 1094 days were also removed. Patients who had experienced prior outcomes were also not considered in this study.

Among the preexisting CDM data, we utilized multiple covariates, such as gender, 5-year age group, Anatomical Therapeutic Chemical (ATC) drug group, SNOMED CT condition group, procedure, measurement value, observation, visit concept count, the CHA2DS2-VASc (congestive heart failure, arterial hypertension, age > 75 years, diabetes mellitus, stroke/transient ischemic attack, vascular disease, age 65–74 years, sex category) score, diabetes complications severity index (DCSI), and the Charlson comorbidity score. Two different covariate settings were tested to determine which PSG parameters could be selected during the cardio-neuro-metabolic disease prediction. One setting (PSG-only covariates) used only gender, age group, and PSG parameters, and the other (all covariates) used all CDM covariates, including the PSG parameters described above as covariates. The observation time windows of the covariates for short, medium, and long terms were set as prior 7 days, 30 days, and 180 days before the cohort start date, respectively.

Three different machine learning models—Lasso Logistic Regression (Lasso), Gradient Boosting Machine (GBM), and Random Forest (RF)—were developed using 25% of the total data for training and 75% for testing. Hyper-parameter training was performed using five-fold cross-validation on the training set. PatientLevelPrediction R package^15^ version 4.0.5 was used for this purpose.

To evaluate the models, model discrimination was assessed using the area under the receiver operating characteristic curve (AUC).

## Results

### Conversion results of PSG parameters into OMOP CDM concepts

We converted data from a total of 11,392 tests corresponding to 11,797 sleep studies into the OMOP CDM format. These included 7,191 full-night PSGs, 2,725 split-night PSGs, 1,474 CPAP titration PSGs, and 407 MSLTs. Among the PSG test results stored in EHR, the conversion target parameters converted into CDM are presented in Table 1. These included 7 pertaining to body measurements, 7 to sleep summaries, 6 to sleep stages, 16 to respiratory events, 4 to apnea or hypopnea duration, 8 to sleep position, 5 to arousals, 2 to limb movement, 5 to snoring, 8 to oxygen statistics, 1 to continuous positive airway pressure, 2 to questionnaires, 11 to MSLT, 1 to apnea level manometry test, and 3 to Friedman staging. A total of 85 PSG parameter concepts were converted to the *measurement* domain and one to the *observation* domain (Waist/hip ratio). Moreover, 20 (23.3%) PSG codes were mapped to the standard OHDSI vocabulary including LOINC and SNOMED CT, but the remaining 66 (76.7%) could not be mapped and were added as new custom standard concepts.

**Table 1:** Polysomnographic parameters included in the Observational Medical Outcomes Partnership (OMOP) Common Data Model (CDM) transformation.

Category	Polysomnographic parameters
Body measurement	Body height (cm), Body weight (Kg), Body mass index (BMI), Neck circumference (cm), Waist circumference (cm), Hip circumference (cm), Waist/hip ratio
Sleep summary	Sleep efficiency (SE) (%), Sleep latency (SL) (min), Sleep period time (SPT) (min), Total sleep time (TST) (min), Total time analyzed (Time In bed, TIB) (min), Wake time after sleep onset (WASO) (min), REM latency from sleep onset
Sleep stage	% stage 1 Nonrapid eye movement (NREM),% stage 2 NREM,% stage 3 NREM,% stage REM, Time spent during REM (min)
Respiratory events	Respiratory disturbance index (RDI), Apnea hypopnea index (AHI) (/h), Apnea index (AI) (/h), Central apnea index (/h), Mixed apnea index (/h), Obstructive apnea index (/h), Hypopnea index (HI) (/h), Hypopnea Index with oxygen desaturation (/h), Hypopnea Index without oxygen desaturation (/h), AHI during supine (/h), AHI during left lateral (/h), AHI during right lateral (/h), AHI during prone (/h), AHI during NREM (/h), AHI during REM (/h), Respiratory effort-related arousal (RERA)
Duration of apnea or hypopnea	Longest apnea duration (second), Mean apnea duration (second), Mean hypopnea duration (second), Mean total apnea and hypopnea duration (second)
Sleep position	Time spent during Supine position (min), % Time spent during Supine position (%), Time spent during Left Lateral position (min), % Time spent during Left Lateral position (%), Time spent during Right Lateral position (min), % Time spent during Right Lateral position (%), Time spent during Prone position (min), % Time spent during Prone position (%)
Arousal	Number of awakenings, Respiratory arousal, Spontaneous arousal, LM with arousals (/h), Periodic limb movement (PLM) arousal
Limb movement	Limb movement index (/h), Periodic limb movement index (PLMI)
Snoring	Average snoring episode duration (min), Longest snoring episode (min), Number of snoring episodes, Snoring percent time (%), Snoring time (min)
Oxygen statistics	%Time of saturation < 60%, %Time of saturation < 70%, %Time of saturation < 80%, %Time of saturation < 90%, Waking oxygen saturation (%), Average oxygen saturation during sleep (%), Lowest oxygen saturation (%), Oxygen desaturation index (ODI)
CPAP pressure	Titrated pressure (cmH2O)
Questionnaire	Epworth sleepiness scale, Pittsburgh sleep quality index
Multiple sleep latency test	REM latency #1 (min), REM latency #2 (min), REM latency #3 (min), REM latency #4 (min), REM latency #5 (min), Sleep latency #1 (min), Sleep latency #2 (min), Sleep latency #3 (min), Sleep latency #4 (min), Sleep latency #5 (min), Mean sleep latency (min)
Apnea level manometry test	% Retroglossal obstruction
Friedman staging	Tonsil grade, Mallampati grade, Friedman stage

### Characteristics of PSG data

The overall characteristics of the total sleep studies that were converted into OMOP CDM are presented in Table 2. Out of an aggregate of 11,392 sleep tests, 8363 (73.4%) tests were conducted on male patients and 3029 (26.6%) on female patients. There was an average of 1.2 tests per person. Tests of patients aged 40–49 years, 50–59 years, and 60–69 years accounted for approximately 65% of the total number of tests. The number of sleep studies conducted each year exhibited a progressive increment. The prevalence of AHI < 5, mild OSA (5 ≤ AHI < 15), moderate OSA (15 ≤ AHI < 30) and severe OSA (30 ≤ AHI) was 28.5%, 23.8%, 19.3% and 28.4%, respectively. The basic statistics of the associated PSG parameters are provided in Supplementary Table S5.

**Table 2:** Demographic characteristics of total sleep tests that were converted into OMOP CDM. The sleep tests from February 2004 to June 2019 were extracted, transformed, and loaded into the OMOP CDM.

Characteristics	Number of records: n (%)	Number of persons: n
Total	11,392	9577
**Gender**		
Male	8363 (73.4)	6829
Female	3029 (26.6)	2748
**Age group**		
< = 9	205 (1.8)	190
10 s	385 (3.4)	368
20 s	565 (5.0)	528
30 s	1229 (10.8)	1063
40 s	2230 (19.6)	1833
50 s	2849 (25.0)	2355
60 s	2348 (20.6)	2016
70 s	1226 (10.8)	1065
80 s	346 (3.0)	313
90 s	9 (0.1)	8
**Year of the sleep study**		
2004	319 (2.8)	288
2005	458 (4)	398
2006	546 (4.8)	495
2007	702 (6.2)	600
2008	639 (5.6)	547
2009	605 (5.3)	528
2010	604 (5.3)	523
2011	647 (5.7)	549
2012	677 (5.9)	582
2013	685 (6)	600
2014	860 (7.5)	751
2015	1014 (8.9)	862
2016	1067 (9.4)	972
2017	1023 (9)	958
2018	1035 (9.1)	1010
2019	511 (4.5)	508
OSA severity levels*	11,250	
AHI < 5	3209 (28.5)	3156
Mild OSA (5 ≤ AHI < 15),	2681 (23.8)	2622
Moderate OSA (15 ≤ AHI < 30)	2167 (19.3)	2091
Severe OSA (AHI30)	3193 (28.5)	3001

*The prevalence of OSA severity levels were calculated based on Apnea Hypopnea Index (AHI) for only records with AHI values.

### Performance of the prediction models

**Figure 2:**
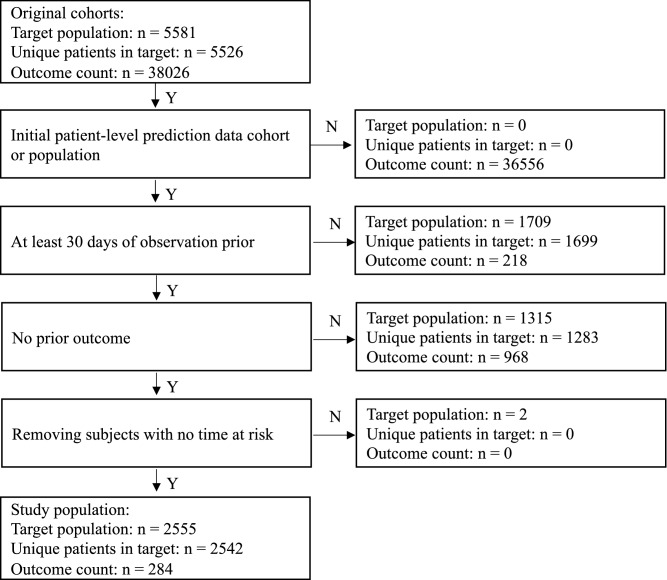
The attrition for the model development at the best performance setting of prediction.

Corresponding to the best performance setting of each prediction models, the number of people eligible for inclusion into the target population, the outcome count, and the number of people lost due to each inclusion step are illustrated in Fig. 2. The target population comprising 5581 full-night PSG tests of patients 18 years or older was reduced to a population comprising 2555 tests of 2542 patients. The outcome rate of cardio-neuro-metabolic disease was observed to be 11.1%.

The AUC performance of the prediction feasibility test based on CDM data achieved an 0.751(0.693–0.810) for the RF model with all covariates. The performance results corresponding to each set of configurations are listed in Table 3. All three models—RF, GBM, and Lasso—performed better when all parameters, such as condition, drug, measurement, and comorbidity score, were utilized as CDM data along with PSG, rather than only the PSG parameters.

**Table 3:** Prediction model performance for test data set. All covariates setting used all OMOP CDM variables including polysomnography parameter concepts, and PSG only covariates used only gender, age group, and polysomnography parameter concepts for developing and training the prediction model.

Covariate setting	Model	Target size (Test)	Outcome count (Test)	Outcome rate (%)	AUC	AUPRC
All covariates	Random forest	639	71	11.11	0.751	0.289
	Gradient boosting machine	483	56	11.59	0.700	0.250
	Lasso Logistic Regression	640	71	11.09	0.672	0.212
PSG only covariates	Random forest	638	71	11.13	0.654	0.213
	Gradient boosting machine	437	50	11.44	0.630	0.170
	Lasso Logistic Regression	482	56	11.62	0.598	0.164

*AUC* area under the receiver operating characteristic curve, *AUPRC* area under the precision recall curve.

The top 20 covariates selected from the RF are presented in Table 4. Among them, 11 were PSG parameters, for example, AHI during right lateral (/h), central apnea index (/h), waking oxygen saturation (%), and snoring time (min). The top 20 covariates selected from the other models are included in Supplementary Table S6.

**Table 4:** Top 20 predictors selected from random forest model. The polysomnography parameters are indicated in bold.

No	Covariate name	Importance	Covariate mean with outcome	Covariate mean with no outcome
1	drug_era group during day -7 through 0 days relative to index: Synthetic antispasmodics, amides with tertiary amines	0.008	0.021	0.001
2	measurement value during day -180 through 0 days relative to index: Triglyceride [Mass/volume] in Serum or Plasma (milligram per deciliter)	0.008	35.158	13.156
3	measurement value during day -180 through 0 days relative to index: Systolic blood pressure (millimeter mercury column)	0.007	43.961	24.646
4	**measurement value during day -30 through 0 days relative to index: AHI during right lateral (/h) (per hour)**	0.006	11.496	7.281
5	measurement value during day -30 through 0 days relative to index: Gamma glutamyl transferase [Enzymatic activity/volume] in Serum or Plasma (unit per liter)	0.006	5.236	1.530
6	measurement value during day -180 through 0 days relative to index: Diastolic blood pressure (millimeter mercury column)	0.006	25.845	14.697
7	drug_era group during day -7 through 0 days relative to index: tiropramide	0.006	0.021	0.001
8	**measurement value during day -180 through 0 days relative to index: AHI during right lateral (/h) (per hour)**	0.006	11.496	7.281
9	**measurement value during day -180 through 0 days relative to index: Central apnea index (/h) (per hour)**	0.006	1.040	0.394
10	**measurement value during day -7 through 0 days relative to index: AHI during right lateral (/h) (per hour)**	0.005	11.496	7.281
11	measurement value during day -7 through 0 days relative to index: Gamma glutamyl transferase [Enzymatic activity/volume] in Serum or Plasma (unit per liter)	0.005	2.923	0.726
12	**measurement value during day -30 through 0 days relative to index: Waking oxygen saturation (%) (percent)**	0.005	74.268	84.735
13	**measurement value during day -7 through 0 days relative to index: Central apnea index (/h) (per hour)**	0.005	1.040	0.394
14	**measurement value during day -30 through 0 days relative to index: Central apnea index (/h) (per hour)**	0.005	1.040	0.394
15	**measurement value during day -7 through 0 days relative to index: Snoring time (min) (minute)**	0.005	111.030	100.081
16	**measurement value during day -7 through 0 days relative to index: AHI during left lateral (/h) (per hour)**	0.004	11.716	7.616
17	**measurement value during day -180 through 0 days relative to index: Respiratory arousal (per hour)**	0.004	22.177	18.561
18	**measurement value during day -7 through 0 days relative to index: Waking oxygen saturation (%) (percent)**	0.004	74.268	84.735
19	drug_era group during day -30 through 0 days relative to index: tiropramide	0.004	0.021	0.002
20	drug_era group during day -180 through 0 days relative to index: tiropramide	0.004	0.028	0.004

## Discussion

To the best of our knowledge, this study represents the first attempt to convert EHR PSG data into ODHSI OMOP CDM, a standard format for health and medical data. Through this study, we successfully converted more than 11,000 PSGs stored in a tertiary hospital EHR into the OMOP CDM version 5.3.1 format. However, we were able to map only approximately 23% of the 86 parameters present within the PSG data to the existing OMOP CDM standard vocabulary, and new custom standard concept names had to be created for the remaining 77% of the parameters. The method used to create the new custom standard concept can be employed when other sites add non-mapping PSG parameters that are not reported in this study.

The most significant advantage of the standardization of EHR data into the CDM format is the speed and efficiency of large-scale analysis afforded to researchers and clinicians using the open-source analysis tools provided by ODHSI^10,12^. Furthermore, due to the inapplicability of OMOP CDM to PSG parameters till date, CDM studies using PSG and MSLT test results, which are the most important tests in sleep medicine, are yet to be conducted. In this context, conversion of PSG results into the CDM format also enables utilization of OHDSI's open-source analytical solutions in clinical studies involving PSG results. In addition, the OMOP CDM format has already been used to standardize a comprehensive collection of EHR data, including diagnostic information, specimen test results, imaging test information, procedure and intervention information, drug exposures, past medical histories, and family histories. Therefore, the standardization procedure attempted in this study enables researchers to conduct robust and scalable analyses involving PSG results in conjunction with pre-CDM-converted large-scale EHR data. Collaborative research across a growing number of sites participating in the standardized CDM network is expected to lead to higher performance in population-level estimation and patient-level prediction models that leverage sleep study parameters.

In this study, the performance of the pilot feasibility test in terms of patient-level prediction for cardio-neuro-metabolic disease exhibited a significant improvement when the entire EHR data along with PSG was used, rather than solely the PSG data. This suggests the feasibility of utilizing all EHR data in the OMOP CDM format via CDM conversion of PSG data.

OSA is a broad-spectrum disease with several different subgroups or phenotypes, and each OSA phenotype is likely to be manifested with different levels of severity, both clinically and objectively^16^. Previous one-size-fits-all approaches based on apnea–hypopnea index suffered from insufficient consideration of these diverse phenotypic subtypes of OSA due to the imperfection of the apnea–hypopnea index as a diagnostic metric with respect to OSA-related symptoms and outcomes^17^. Several studies have demonstrated that each OSA phenotype exhibits different characteristics and varying risks of disease outcomes^16,18^. The most important data included in these studies were various metrics of PSG, including all the PSG results, which enabled the classification of OSA into various phenotypes via the phenotyping technique. One study that attempted a structured, data-driven approach based on multiple PSG features of approximately 2,000 OSA patients was able to identify seven subgroups (phenotypes). The aforementioned study also revealed that a unique phenotype that may have been missed during conventional OSA severity classification based on a single metric—apnea hypopnea index—could account for the risk of cardiovascular outcome more effectively^19^. In our previous study, we also identified four clusters based on various PSG features and there was a significant difference in disease outcome among the clusters, and such a difference could not be found in the standard classification of OSA based only on AHI severity^20^. Moreover, these characteristic phenotypes may exhibit different patterns depending on race, country, or individual. Therefore, to improve the ability to predict adverse OSA outcomes for a population or an individual, simply having a large number of PSGs is not sufficient—it is necessary to acquire PSGs across various data sources. Therefore, it is advantageous to use standardized data such as OMOP CDM to increase the reproducibility and statistical significance of the analyses. The conversion of data into the OMOP CDM format enables ATLAS, OHDSI's open-source analytic solution, to generate queries that can set the aforementioned OSA phenotypes as target cohorts and queries that can set OSA complications to be predicted as the outcome cohort. This enables verification of the reproducibility of outcome predictions of OSA phenotyping through analysis of the dataset including PSG with the same queries in multiple sleep centers where PSG-CDM standardization has been completed. In addition to the analysis of large-scale PSG data, the clinical relevance of the OSA phenotypes across various populations by region and race will be able to be also verified.

With the increase in CDM conversion of EHR data across medical institutions, research based on CDM-format datasets is expected to be pursued in various fields. However, unlike the CDM conversion of data such as clinical diagnosis results, laboratory sample test results, and drug exposure data, the CDM conversion of medical data based on patient-generated signals, including PSG, is still insufficient. Therefore, till date, CDM-based research has been actively conducted in fields where conversion to the pre-existing standard vocabulary is feasible. Domains where CDM research is most active include pharmacovigilance^21–23^ and pharmacoepidemiology^24^. For example, a study assessing anti-seizure drug-related adverse reactions in 1344 target epilepsy cohorts determined that the detection rate of the adverse drug reaction based on CDM-format data was comparable to previously published results obtained using traditional data analysis techniques^21^. In addition, it is possible to implement various designs of research by constructing a target cohort corresponding to a study entry population and an outcome cohort corresponding to a disease outcome population^25,26^. Examples include a prognostic model validation study predicting hemorrhagic transformation of acute ischemic stroke within a CDM dataset of more than 600,000 patients via the OHDSI international network^25^, and a survival analysis study using 115 variables in 346 patients diagnosed with intrahepatic cholangiocarcinoma^26^.

Despite the significant implications, the present study has certain limitations. First, the rate of correspondence between ODHSI's standard OMOP CDM concepts and PSG parameters was as low as approximately 20%. This can be attributed to the fact that the pre-existing OMOP CDM standard vocabulary does not reflect all of the approximately 80 PSG variables considered in this study. The custom standard vocabulary developed to address this limitation in this study is expected to contribute to future studies that utilize PSG parameters in CDM-based EHR studies. When creating the custom concepts, we made it easy to find all PSG parameters by defining the relationship to the PSG order. For concepts that may have varying definitions, the definition of the concept is provided as metadata. For concepts (e.g., %Time of saturation < 60%, %Time of saturation < 70%) in which multiple criteria can exist, a concept was created in a way that has individual *concept_id*s. Since the *MEASUREMENT* table does not have a modifier attribute, it would be the best practice to create individual concepts for them. By doing this, the meaning of new concepts can be clarified. As the basic PSG parameters of the PSG recording systems of the various vendors are similar, we think other institutions will also be able to apply the new concept proposed in this study. In addition, we look forward to adding the new concepts to OHDSI's standard vocabulary. Second, in South Korea, insurance for CPAP began in July 2018; before then, it had been recorded in a different form of EHR rather than an order. Thus, in this study, only CPAP orders after July 2018 were converted to CDM and can be used as predictors for the pilot prediction models. There could be an issue where information on orders for CPAP, which may be an important variable in predicting cardio-neuro-metabolic disease, is not complete. However, as the purpose of this study was only to demonstrate the pilot feasibility of the prediction model using CDM including PSG data, predictors should be considered more elaborately when developing a prediction model in the future. Third, different sleep centers represent PSG databases in EHRs in different ways. Many centers store PSG results in EHR as an image file, or simply record OSA severity in a report format. Therefore, significant implementation effort and time is required to extract, transform, and load the PSG results into the CDM format. Furthermore, different levels of digitization of PSG data in different hospitals may cause concerns regarding the different levels of CDM conversion from PSG parameters. However, with the increase in CDM studies including PSG parameters, the electronic representation of PSG data in the EHR system is expected to be facilitated across hospitals. Finally, conversion of data into the CDM format is time-consuming, requiring a substantial amount of resources, in addition to the fundamental requirement of collecting native source data. The need to code subsets of data manually may limit conversion efforts. However, once the native data are converted to the CDM format, EHR systems in the network will be able to use the same queries to identify cohorts. Thus, conversion to CDM is expected to minimize the effort required to develop cohorts and analyze results across multiple sites.

The harmonization across different sites requires collaborative efforts from multidisciplinary experts, including clinical domain experts, terminology experts, and engineers from various sites. When other sites try to map their own PSG data, efforts should be made to use and propose the same vocabulary and the same concept as much as possible by using the mapping result proposed in this study or by participating in the OHDSI community. As the standard terminology for PSG data has not yet been established internationally, if a specific ontology for sleep study can be proposed as OHDSI vocabulary by reviewing previous efforts, such as the Sleep Domain Ontology and the National Sleep Research Resource, it is expected to be helpful in the conversion and expansion of CDM by other sites.

## Conclusions

The Observational Medical Outcomes Partnership (OMOP) Common Data Model (CDM) is a standard data format and has been applied to various EHR databases. However, its application to PSG data has not been attempted till date. To the best of our knowledge, this study represents the first attempt to transform PSG data into the OMOP CDM format. Well-defined large-volume OMOP CDM databases of PSG data can potentially enable the identification of clinically relevant OSA phenotypes, estimation of disease outcomes at the population level and prediction of outcomes at the patient-level. We expect the CDM mapping and CDM custom vocabulary of the PSG proposed in this study to contribute to the CDM conversion of PSG databases and future studies leveraging such databases.

## Supplementary Information

Supplementary Information 1.

Supplementary Information 2.

Supplementary Information 3.

## Data Availability

CDM data are designed to support a distributed research network. Thus, access to the data is restricted on internal private networks. Therefore, the data are not publicly available.
