# Supplementary material for: Transforming electronic health record polysomnographic data into the Observational Medical Outcome Partnership's Common Data Model: a pilot feasibility study
Source: Sci Rep. 2021 Mar 29;11:7013. doi: 10.1038/s41598-021-86564-w (PMC8007756; doi:10.1038/s41598-021-86564-w)
Supplement: Supplementary file 1 — Supplementary Information 1. [file 41598_2021_86564_MOESM1_ESM.docx]

**Transforming electronic health record polysomnographic data into the Observational Medical Outcome Partnership's Common Data Model: A Pilot Feasibility Study**

Jeong-Whun Kim^1,2^, Seok Kim^3^, Borim Ryu^3^, Wongeun Song^3^, Ho-young Lee^3^, Sooyoung Yoo^3*^

^1^Department of Otorhinolaryngology, Seoul National University College of Medicine, Seoul;

^2^Department of Otorhinolaryngology, Seoul National University Bundang Hospital, Seongnam;

^3^Office of eHealth Research and Business, Seoul National University Bundang Hospital, Seongnam, South Korea

**Corresponding author:**

^*^Sooyoung Yoo, Ph.D.

Office of eHealth Research and Businesses, 172, Dolma-ro, Bundang-gu, Seongnam-si, Gyeonggi-do 13605, Republic of Korea

E-mail: yoosoo0@snubh.org

Tel: +82-31-787- 8980

**Supplementary Table S1.** Conversion of polysomnographic parameters into the Observational Medical Outcomes Partnership (OMOP) Common Data Model (CDM).

| **Category** | **Polysomnographic parameters** | **CDM concept name** | **Domain** | **Vocabulary** | **Concept ID** |
| --- | --- | --- | --- | --- | --- |
| Body measurement | Body height (cm) | Body height | Measurement | LOINC | 3036277 |
|  | Body weight (Kg) | Body weight | Measurement | LOINC | 3025315 |
|  | Body mass index (BMI) | Body mass index (BMI) | Measurement | LOINC | 3038553 |
|  | Neck circumference (cm) | Circumference Neck | Measurement | LOINC | 40759195 |
|  | Waist circumference (cm) | Waist Circumference at umbilicus by Tape measure | Measurement | LOINC | 3016258 |
|  | Hip circumference (cm) | Circumference.at maximal protrusion of gluteus muscles Pelvis | Measurement | LOINC | 40759184 |
|  | Waist/hip ratio | Waist/hip ratio | Observation | SNOMED | 4087501 |
| Sleep summary | Sleep efficiency (SE) (%) | Sleep efficiency (SE) (%) | Measurement | custom standard | 2050117591 |
|  | Sleep latency (SL) (min) | Sleep latency (SL) (min) | Measurement | custom standard | 2050117592 |
|  | Sleep period time (SPT) (min) | Sleep period time (SPT) (min) | Measurement | custom standard | 2050117593 |
|  | Total sleep time (TST) (min) | Total sleep time (TST) (min) | Measurement | custom standard | 2050117594 |
|  | Total time analyzed (Time In bed, TIB) (min) | Total time analyzed (Time In bed, TIB) (min) | Measurement | custom standard | 2050117595 |
|  | Wake time after sleep onset (WASO) (min) | Wake time after sleep onset (WASO) (min) | Measurement | custom standard | 2050117596 |
|  | REM latency from sleep onset (min) | REM latency from sleep onset (min) | Measurement | custom standard | 2050117590 |
| Sleep stage | % stage 1 Nonrapid eye movement (NREM) | % stage 1 Nonrapid eye movement (NREM) | Measurement | custom standard | 2050117583 |
|  | % stage 2 NREM | % stage 2 NREM | Measurement | custom standard | 2050117584 |
|  | % stage 3 NREM | % stage 3 NREM | Measurement | custom standard | 2050117585 |
|  | % stage REM | % stage REM | Measurement | custom standard | 2050117587 |
|  | Time spent during REM (min) | Time spent during REM (min) | Measurement | custom standard | 2050117575 |
|  | Time spent during NREM (min) | Time spent during NREM (min) | Measurement | custom standard | 2050117570 |
| Respiratory events | Respiratory disturbance index (RDI) (/h) | Respiratory disturbance index (RDI) | Measurement | LOINC | 1175351 |
|  | Apnea hypopnea index (AHI) (/h) | Apnea Hypopnea Index | Measurement | LOINC | 37396400 |
|  | Apnea index (AI) (/h) | Apnea index | Measurement | LOINC | 1175153 |
|  | Central apnea index (/h) | Central apnea index (/h) | Measurement | custom standard | 2050117560 |
|  | Mixed apnea index (/h) | Mixed apnea index (/h) | Measurement | custom standard | 2050117564 |
|  | Obstructive apnea index (/h) | Obstructive apnea index (/h) | Measurement | custom standard | 2050117565 |
|  | Hypopnea index (HI) (/h) | Hypopnea index | Measurement | LOINC | 1175647 |
|  | Hypopnea Index with oxygen desaturation (/h) | Hypopnea Index with oxygen desaturation (/h) | Measurement | custom standard | 2050117562 |
|  | Hypopnea Index without oxygen desaturation (/h) | Hypopnea Index without oxygen desaturation (/h) | Measurement | custom standard | 2050117563 |
|  | AHI during supine (/h) | AHI during supine (/h) | Measurement | custom standard | 2050117579 |
|  | AHI during left lateral (/h) | AHI during left lateral (/h) | Measurement | custom standard | 2050117566 |
|  | AHI during right lateral (/h) | AHI during right lateral (/h) | Measurement | custom standard | 2050117576 |
|  | AHI during prone (/h) | AHI during prone (/h) | Measurement | custom standard | 2050117571 |
|  | AHI during NREM (/h) | AHI during NREM (/h) | Measurement | custom standard | 2050117569 |
|  | AHI during REM (/h) | AHI during REM (/h) | Measurement | custom standard | 2050117574 |
|  | Respiratory effort-related arousal (RERA) (/h) | Respiratory effort-related arousal index | Measurement | LOINC | 1175863 |
| Duration of apnea or hypopnea | Longest apnea duration (second) | Longest apnea duration (second) | Measurement | custom standard | 2050117534 |
|  | Mean apnea duration (second) | Mean apnea duration (second) | Measurement | custom standard | 2050117535 |
|  | Mean hypopnea duration (second) | Mean hypopnea duration (second) | Measurement | custom standard | 2050117536 |
|  | Mean total apnea and hypopnea duration (second) | Mean total apnea and hypopnea duration (second) | Measurement | custom standard | 2050117537 |
| Sleep position | Time spent during Supine position (min) | Time spent during Supine position (min) | Measurement | custom standard | 2050117581 |
|  | % Time spent during Supine position (%) | % Time spent during Supine position (%) | Measurement | custom standard | 2050117580 |
|  | Time spent during Left Lateral position (min) | Time spent during Left Lateral position (min) | Measurement | custom standard | 2050117568 |
|  | % Time spent during Left Lateral position (%) | % Time spent during Left Lateral position (%) | Measurement | custom standard | 2050117567 |
|  | Time spent during Right Lateral position (min) | Time spent during Right Lateral position (min) | Measurement | custom standard | 2050117578 |
|  | % Time spent during Right Lateral position (%) | % Time spent during Right Lateral position (%) | Measurement | custom standard | 2050117577 |
|  | Time spent during Prone position (min) | Time spent during Prone position (min) | Measurement | custom standard | 2050117573 |
|  | % Time spent during Prone position (%) | % Time spent during Prone position (%) | Measurement | custom standard | 2050117572 |
| Arousal | Number of awakenings | Number of awakenings | Measurement | custom standard | 2050117588 |
|  | Respiratory arousal (/h) | Respiratory arousal (/h) | Measurement | custom standard | 2050117524 |
|  | Spontaneous arousal (/h) | Spontaneous arousal (/h) | Measurement | custom standard | 2050117525 |
|  | LM with arousals (/h) | LM with arousals (/h) | Measurement | custom standard | 2050117554 |
|  | Periodic limb movement (PLM) arousal (/h) | Periodic limb movement (PLM) arousal (/h) | Measurement | custom standard | 2050117523 |
| Limb movement | Limb movement index (/h) | Limb movement index (/h) | Measurement | custom standard | 2050117553 |
|  | Periodic limb movement index (PLMI) | Periodic limb movement index (PLMI) | Measurement | custom standard | 2050117555 |
| Snoring | Average snoring episode duration (min) | Average snoring episode duration (min) | Measurement | custom standard | 2050117597 |
|  | Longest snoring episode (min) | Longest snoring episode (min) | Measurement | custom standard | 2050117598 |
|  | Number of snoring episodes | Number of snoring episodes | Measurement | custom standard | 2050117599 |
|  | Snoring percent time (%) | Snoring percent time (%) | Measurement | custom standard | 2050117600 |
|  | Snoring time (min) | Snoring time (min) | Measurement | custom standard | 2050117601 |
| Oxygen statistics | %Time of saturation < 60% | %Time of saturation < 60% | Measurement | custom standard | 2050117602 |
|  | %Time of saturation < 70% | %Time of saturation < 70% | Measurement | custom standard | 2050117603 |
|  | %Time of saturation < 80% | %Time of saturation < 80% | Measurement | custom standard | 2050117604 |
|  | %Time of saturation < 90% | %Time of saturation < 90% | Measurement | custom standard | 2050117605 |
|  | Waking oxygen saturation (%) | Waking oxygen saturation (%) | Measurement | custom standard | 2050117609 |
|  | Average oxygen saturation during sleep (%) | Blood oxygen saturation | Measurement | SNOMED | 4310328 |
|  | Lowest oxygen saturation (%) | Lowest oxygen saturation (%) | Measurement | custom standard | 2050117607 |
|  | Oxygen desaturation index (ODI) | Oxygen desaturation index | Measurement | LOINC | 1175603 |
| CPAP pressure | Titrated pressure (cmH2O) | Continuous positive airway pressure Respiratory system | Measurement | LOINC | 3035167 |
| Questionnaire | Epworth sleepiness scale | Epworth Sleepiness Scale | Measurement | SNOMED | 46273534 |
|  | Pittsburgh sleep quality index | Pittsburgh sleep quality index | Measurement | SNOMED | 44783153 |
| Multiple sleep latency test | REM latency #1 (min) | REM latency #1 (min) | Measurement | custom standard | 2050117542 |
|  | REM latency #2 (min) | REM latency #2 (min) | Measurement | custom standard | 2050117543 |
|  | REM latency #3 (min) | REM latency #3 (min) | Measurement | custom standard | 2050117544 |
|  | REM latency #4 (min) | REM latency #4 (min) | Measurement | custom standard | 2050117545 |
|  | REM latency #5 (min) | REM latency #5 (min) | Measurement | custom standard | 2050117546 |
|  | Sleep latency #1 (min) | Sleep latency #1 (min) | Measurement | custom standard | 2050117547 |
|  | Sleep latency #2 (min) | Sleep latency #2 (min) | Measurement | custom standard | 2050117548 |
|  | Sleep latency #3 (min) | Sleep latency #3 (min) | Measurement | custom standard | 2050117549 |
|  | Sleep latency #4 (min) | Sleep latency #4 (min) | Measurement | custom standard | 2050117550 |
|  | Sleep latency #5 (min) | Sleep latency #5 (min) | Measurement | custom standard | 2050117551 |
|  | Mean sleep latency (min) | Mean sleep latency (min) | Measurement | custom standard | 2050117552 |
| Apnea level manometry test | % Retroglossal obstruction | % Retroglossal obstruction | Measurement | custom standard | 2050117522 |
| Friedman staging | Tonsil grade | Physical findings of Tonsil | Measurement | LOINC | 3005810 |
|  | Mallampati grade | Mallampati score | Measurement | SNOMED | 4165278 |
|  | Friedman stage | Friedman classification | Measurement | SNOMED | 764570 |

**Supplementary Table S2.** Definitions for populating metadata

| **Name** | **Value_as_string** |
| --- | --- |
| Sleep study device name | Embla N 7000 (Embla, Reykjavik, Iceland) recording system |
| Sleep study scoring software name | Embla RemLogic PSG Software (Embla, ON, Canada) |
| Hypopnea index | A reduction in the airflow by 50% or more lasting at least 10 s, or the accompaniment of airflow reduction by arousal or an oxygen desaturation by 4% or more |
| Sleep efficiency (SE) (%) | Total sleep time/total time in bed×100 |
| Sleep latency (SL) (min) | Time spent from lights out to first epoch of any sleep |
| Sleep period time (SPT) (min) | Duration of time from sleep onset to final awakening |
| Total sleep time (TST) (min) | Total of all REM and NREM sleep time |
| Total time analyzed (Time In bed, TIB) (min) | Total amount of time analyzed in bed, total recording time |
| Wake time after sleep onset (WASO) (min) | Time in bed – Sleep latency – Total sleep time |
| REM latency from sleep onset (min) | Time spent from sleep onset to first epoch of sleep stage REM |
| % stage 1 Nonrapid eye movement (NREM) | stage 1 NREM/total sleep time×100 |
| % stage 2 NREM | % stage 2 NREM/total sleep time×100 |
| % stage 3 NREM | % stage 3 NREM/total sleep time×100 |
| % stage REM | % stage REM/total sleep time×100 |
| Time spent during REM (min) | Time spent during REM sleep stage |
| Time spent during NREM (min) | Time spent during NREM sleep stages |
| Central apnea index (/h) | (Number of central apneas× 60) / TST |
| Mixed apnea index (/h) | (Number of mixed apneas× 60) / TST |
| Obstructive apnea index (/h) | (Number of obstructive apneas× 60) / TST |
| Hypopnea Index with oxygen desaturation (/h) | (Number of hypopneas with oxygen desaturation≥4%× 60) / TST |
| Hypopnea Index without oxygen desaturation (/h) | (Number of hypopneas without oxygen desaturation × 60) / TST |
| AHI during supine (/h) | (Number of apneas + hypopneas during supine sleep position× 60) / time spent during supine position in min |
| AHI during left lateral (/h) | (Number of apneas + hypopneas during left lateral sleep position× 60) / time spent during left lateral position in min |
| AHI during right lateral (/h) | (Number of apneas + hypopneas during right lateral sleep position× 60) / time spent during right lateral position in min |
| AHI during prone (/h) | (Number of apneas + hypopneas during prone sleep position× 60) / time spent during prone position in min |
| AHI during NREM (/h) | (Number of apneas + hypopneas during NREM sleep stage× 60) / time spent during NREM sleep stage in min |
| AHI during REM (/h) | (Number of apneas + hypopneas during REM sleep stage× 60) / time spent during REM sleep stage in min |
| Longest apnea duration (second) | Longest apnea duration in second |
| Mean apnea duration (second) | Mean duration of apneas in second |
| Mean hypopnea duration (second) | Mean duration of hypopneas in second |
| Mean total apnea and hypopnea duration (second) | Mean duration of apneas and hypopnea in second |
| Time spent during Supine position (min) | Time spent during supine sleep position in min |
| % Time spent during Supine position (%) | Time spent during supine sleep position/TST×100 |
| Time spent during Left Lateral position (min) | Time spent during left lateral sleep position in min |
| % Time spent during Left Lateral position (%) | Time spent during left lateral sleep position/TST×100 |
| Time spent during Right Lateral position (min) | Time spent during right lateral position in min |
| % Time spent during Right Lateral position (%) | Time spent during right lateral sleep position/TST×100 |
| Time spent during Prone position (min) | Time spent during prone position in min |
| % Time spent during Prone position (%) | Time spent during prone sleep position/TST×100 |
| Number of awakenings | Number of awakenings |
| Respiratory arousal (/h) | (Number of respiratory arousals×60) / TST |
| Spontaneous arousal (/h) | (Number of spontaneous arousals×60) / TST |
| LM with arousals (/h) | (Number of LM arousals×60) / TST |
| Periodic limb movement (PLM) arousal (/h) | (Number of PLM arousals×60) / TST |
| Limb movement index (/h) | (Number of LMs×60) / TST |
| Periodic limb movement index (PLMI) (/h) | (Number of PLM×60) / TST |
| Average snoring episode duration (min) | Average duration in min of snoring episodes |
| Longest snoring episode (min) | Longest snoring episode in min |
| Number of snoring episodes | Number of snoring episodes |
| Snoring percent time (%) | Total duration of snoring episodes in min/TST×100 |
| Snoring time (min) | Total duration of snoring episodes |
| %Time of saturation < 60% | (Time of oxygen saturation < 60% in min)/TST×100 |
| %Time of saturation < 70% | (Time of oxygen saturation < 70% in min)/TST×100 |
| %Time of saturation < 80% | (Time of oxygen saturation < 80% in min)/TST×100 |
| %Time of saturation < 90% | (Time of oxygen saturation < 90% in min)/TST×100 |
| Waking oxygen saturation (%) | Average oxygen saturation during waking time |
| Lowest oxygen saturation (%) | Lowest oxygen saturation |
| REM latency #1 (min) | Time spent from sleep onset to first epoch of sleep stage REM in the first round of sleep for multiple sleep latency test (MSLT) |
| REM latency #2 (min) | Time spent from sleep onset to first epoch of sleep stage REM in the second round of sleep for multiple sleep latency test (MSLT) |
| REM latency #3 (min) | Time spent from sleep onset to first epoch of sleep stage REM in the third round of sleep for multiple sleep latency test (MSLT) |
| REM latency #4 (min) | Time spent from sleep onset to first epoch of sleep stage REM in the fourth round of sleep for multiple sleep latency test (MSLT) |
| REM latency #5 (min) | Time spent from sleep onset to first epoch of sleep stage REM in the fifth round of sleep for multiple sleep latency test (MSLT) |
| Sleep latency #1 (min) | Time spent from lights out to first epoch of any sleep in the first round of sleep for multiple sleep latency test (MSLT) |
| Sleep latency #2 (min) | Time spent from lights out to first epoch of any sleep in the second round of sleep for multiple sleep latency test (MSLT) |
| Sleep latency #3 (min) | Time spent from lights out to first epoch of any sleep in the third round of sleep for multiple sleep latency test (MSLT) |
| Sleep latency #4 (min) | Time spent from lights out to first epoch of any sleep in the fourth round of sleep for multiple sleep latency test (MSLT) |
| Sleep latency #5 (min) | Time spent from lights out to first epoch of any sleep in the fifth round of sleep for multiple sleep latency test (MSLT) |
| Mean sleep latency (min) | Average time in min of sleep latencies in multiple sleep latency test (MSLT) |
| % Retroglossal obstruction | (Number of retroglossal obstruction events/number of retropalatal obstruction events + number of retroglossal obstruction events)×100 |

**Supplementary Table S3.** Data quality check rules for polysomnography result data

| **Category** | **Polysomnographic parameters** | **Rules** | **Filtered record count** | **# of records remaining** |
| --- | --- | --- | --- | --- |
| All | - | There should be no NULL data. |  |  |
|  |  | If the unit is percent, it cannot exceed 100. | 4 |  |
|  |  | If the unit is minute, the value cannot exceed total time analyzed. | 23 |  |
| Body measurement | Body height (cm) | 1. Body height cannot be less than 20cm  2. Body height cannot be more than 300cm  3. Under 15 years of age, body height cannot be more than 200cm  4. Body height cannot be less than 50cm in 3 years old or older  5. Body height cannot be less than 100cm in age 16 or older. | 3 | 11336 |
|  | Body weight (Kg) | 1. Body weight cannot be less than 0.2kg  2. Body weight cannot exceed 300kg.  3. Under 15 years of age, body weight cannot exceed 150kg.  4. Body weight cannot be less than 10kg in 16 years old or older. | 1 | 11350 |
|  | Body mass index (BMI) | 1. BMI cannot exceed 100.  2. BMI cannot be zero. | 3 | 11334 |
|  | Neck circumference (cm) | Neck circumference cannot exceed 100. | 2 | 9614 |
|  | Waist circumference (cm) | Waist circumference cannot exceed 200. | 1 | 9608 |
|  | Hip circumference (cm) | Hip circumference cannot exceed 200. | 1 | 9596 |
|  | Waist/hip ratio | Waist/hip ratio cannot exceed 2. | 47 | 9535 |
| Sleep summary | Sleep latency (SL) (min) | Sleep latency (SL) cannot exceed total sleep time (TST). | 110 | 11271 |
|  | Total sleep time (TST) (min) | Total Sleep Time cannot exceed 720 minutes. | 0 | 11388 |
|  | Total time analyzed (Time In bed, TIB) (min) | 1. Total Time Analyzed cannot be NULL.  2. Total Time Analyzed cannot be over 1000 | 0 | 11392 |
| Respiratory events | AHI during supine (/h) | AHI during supine (/h) cannot exceed 200 | 1 | 9711 |
|  | AHI during NREM (/h) | AHI during NREM (/ h) cannot exceed 200. | 1 | 10667 |
| Duration of apnea or hypopnea | Longest apnea duration (second) | Longest apnea duration (second) cannot exceed 200. | 3 | 10845 |
| Sleep position | Time spent during Supine position (min) | Time spent during Supine position (min) cannot exceed> 600. | 1 | 9685 |
| Arousal | Respiratory arousal | "Respiratory Arousal" should be less than or equal to "Respiratory Disturbance Index (/ h)". | 85 | 10700 |
|  | Periodic limb movement (PLM) arousal | "Periodic Limb Movement (PLM) Arousal" should be less than or equal to "Periodic limb movement index (PLMI)". | 297 | 8311 |
| Snoring | Average snoring episode duration (min) | Average snoring episode duration cannot be greater than longest snoring episode. | 5 | 10641 |
| Oxygen statistics | %Time of saturation < 60% | '% Time of Saturation <60%' cannot be greater than 10. | 1 | 706 |
|  | Waking oxygen saturation (%) | Waking oxygen saturation must be greater than 30. | 2 | 9303 |
|  | Average oxygen saturation during sleep (%) | Blood oxygen saturation must be greater than 60. | 1 | 11356 |
|  | Lowest oxygen saturation (%) | Lowest oxygen saturation should be greater than 50. | 45 | 11300 |
| CPAP pressure | Titrated pressure (cmH2O) | Titrated pressure cannot exceed 30. | 3 | 2363 |
| Questionnaire | Epworth sleepiness scale | Epworth sleepiness scale cannot exceed 24. | 4 | 7850 |
|  | Pittsburgh sleep quality index | Pittsburgh sleep quality index cannot exceed 21. | 6 | 7740 |
| Friedman staging | Tonsil grade | Physical findings of Tonsil cannot exceed 4. | 2 | 3429 |
|  | Mallampati grade | Mallampati score cannot exceed 4. | 2 | 3245 |
|  | Friedman stage | Friedman classification cannot exceed 4. | 3 | 3158 |

**Supplementary Table S4.** ICD-10 codes for defining cardio-neuro-metabolic disease considered in this study

| **Disease** | **ICD-10 Code** |
| --- | --- |
| Hypertension | I10-13 |
| Dyslipidemia | E78 |
| Diabetes Mellitus Type 2 | E11-E14 |
| Ischemic Heart Disease | I20-I25 |
| Atrial Fibrillation and Flutter | I48 |
| Congestive Heart Failure | I50 |
| Aortic Aneurysm | I71 |
| Cardiomyopathy | I42-I43 |
| Stroke | G45-G46, I60-I63, I67-I69 |
